# Hispolon induces apoptosis in oral squamous cell carcinoma cells through JNK/HO‐1 pathway activation

**DOI:** 10.1111/jcmm.17729

**Published:** 2023-03-27

**Authors:** Wei‐En Yang, Yi‐Tzu Chen, Chun‐Wen Su, Mu‐Kuan Chen, Chia‐Ming Yeh, Yen‐Lin Chen, Meng‐Ying Tsai, Shun‐Fa Yang, Chiao‐Wen Lin

**Affiliations:** ^1^ Department of Medical Research Chung Shan Medical University Hospital Taichung Taiwan; ^2^ Institute of Medicine, Chung Shan Medical University Taichung Taiwan; ^3^ School of Dentistry Chung Shan Medical University Taichung Taiwan; ^4^ Department of Dentistry Chung Shan Medical University Hospital Taichung Taiwan; ^5^ Department of Otorhinolaryngology‐Head and Neck Surgery, Changhua Christian Hospital Changhua Taiwan; ^6^ Oral cancer Research Center, Changhua Christian Hospital Changhua Taiwan; ^7^ Institute of Oral Sciences, Chung Shan Medical University Taichung Taiwan

**Keywords:** apoptosis, heme oxygenase 1, hispolon, JNK1/2, oral squamous cell carcinoma

## Abstract

Oral squamous cell carcinoma (OSCC) has a high recurrence rate and poor prognosis. Hispolon, a polyphenolic compound with antiviral, antioxidant, and anticancer activities, is a potential chemotherapy agent. However, few studies have investigated the anti‐cancer mechanism of hispolon in oral cancer. This present study used the cell viability assay, clonogenic assay, fluorescent nuclear staining, and flow cytometry assay to analyse the apoptosis‐inducing effects of hispolon in OSCC cells. After hispolon treatment, the apoptotic initiators, cleaved caspase‐3, −8, and − 9, were upregulated, whereas the cellular inhibitor of apoptosis protein‐1 (cIAP1) was downregulated. Furthermore, a proteome profile analysis using a human apoptosis array revealed the overexpression of heme oxygenase‐1 (HO‐1) by hispolon, which was determined to be involved in caspase‐dependent apoptosis. Moreover, cotreatment with hispolon and mitogen‐activated protein kinase (MAPK) inhibitors revealed that hispolon induces apoptosis in OSCC cells through activation of the c‐Jun N‐terminal kinase (JNK) pathway and not the extracellular signal‐regulated kinase (ERK) or p38 pathway. These findings indicate that hispolon may exert an anticancer effect on oral cancer cells by upregulating HO‐1 and inducing caspase‐dependent apoptosis by activating the JNK pathway.

## 1 INTRODUCTION

Oral cancer belongs to malignancies that occurred in oral cavity, such as tongue, the upper and lower gums, lips, cheek, and the floor of mouth. Oral squamous cell carcinoma (OSCC) is a major type of oral cancer, which may be associated with various carcinogens present in oral habits such as alcohol, tobacco smoking, and betel quid chewing.^1^, ^2^, ^3^, ^4^, ^5^ OSCC frequently caused cervical lymph node spreading, even distant metastasis.^6^ Due to high recurrence rate, the prognosis of OSCC patients at advance stage remains unfavourable. The main treatment strategies of patients with oral cancer used to be surgery or radiation therapy, or both of them.^7^ Nevertheless, the combination of systemic therapy, such as immunotherapy and chemotherapy, has made significant progress in the clinical treatment of oral cancer patients in the past several years.^8^

Chemotherapy has been extensively given in oral cancer patients with locally advanced disease to retard the tumour growth, the given time may be before or after surgery.^9^ Single or combination usage of chemotherapeutic agents, such as carboplatin, 5‐fluorouracil (5‐FU), cisplatin, and docetaxel, are often managed for oral cancer treatment.^10^, ^11^, ^12^, ^13^ These agents are able to induce sequence of cellular response that influence on tumour cell proliferation and survival. The deficient of apoptosis is the basis of tumour development; therefore, to activatee this programmed cell death become important to development of cancer treatment. The initiation of apoptosis depends on the initiator and effector cysteine‐containing asparte‐specific proteases (caspases), which activate apoptosis signal and execute proteolysis.^14^ Through the death receptor stimulation, the cleaved initiator caspase‐8 subsequently provokes two pathways, the effector caspase‐3/‐7 pathway and mitochondrial pathway via Bid.^15^ The cleaved initiator caspase‐9 also essential for activating apoptosome complex via mitochondrial pathway.^16^ These mechanisms resulted in cellular morphological changes of apoptosis such as membrane blebbing and DNA fragmentation.^17^

Hispolon belongs to a polyphenol compound, which was found in several types of mushrooms such as *Inonotus hispidus*, *Phallus linteus*, and *Phellinus igniarius*.^18^ To date, hispolon has been widely discovered to have the activities of anti‐virus,^18^ anti‐oxidant,^19^ anti‐inflammatory,^20^ hepato‐protective,^21^ and anticancer.^22^ Huang et al. have shown the apoptotic effect of hispolon by modulating ERK pathway on hepatocellular carcinoma cells.^23^ Previously, our study also reported that hispolon suppressed metastasis on cervical cancer cells^24^ and nasopharyngeal carcinoma cells.^25^ Although the anticancer effect of hispolon has been found in various of tumour types, the mechanism of hispolon still lack on OSCC. In our study, we explored the anti‐proliferative effect on OSCC cell lines, including SCC‐9 and HSC‐3 cells, and analysed the underlying mechanism of hispolon‐induced apoptosis. Our study merited to provide the evidence to support the availability of hispolon in chemotherapeutic treatment.

## 2 MATERIALS AND METHODS

### 2.1 Cells and cell culture

Human oral squamous carcinoma cell lines SCC‐9 and HSC‐3 were obtained from American Type Culture Collection (ATCC); human gingival epithelioid cell line Smulow–Glickman (SG) was established by Smulow, J.B. and I. Glickman.^26^ SCC‐9 cell line was cultured in DMEM/F‐12 medium (Gibco) supplemented with nonessential amino acids (NEAA; Life Technologies), 10% foetal bovine serum (FBS; Gibco), 1% penicillin/streptomycin, and 400 ng/mL hydrocortisone. HSC‐3 cell line was cultured in minimal essential medium (MEM) (Gibco) supplemented with 10% FBS and 1% penicillin/streptomycin. SG cell line was cultured in Dulbecco's modified MEM (DMEM) medium supplemented with 10% FBS and 1% penicillin/streptomycin. Cell cultures were maintained at 37°C with 5% CO_2_ in a humidified incubator. For hispolon treatment, 98% purity of hispolon was obtained from Enzo Life Sciences (NY, USA; CAS number: 173933‐40‐9) and dissolved in 100% dimethyl sulfoxide (DMSO) (Sigma Chemical) for stock solution. The stock solution was added to the culture medium to establish the test concentrations (0–200 μM) for indicate time.

### 2.2 Cell viability assay

The SCC‐9, HSC‐3 and SG cell lines were seeded in 24‐well plates in 80–90% confluent, then vehicle control or hispolon were treated for 24 h. By adding 3‐(4,5‐dimethylthiazol‐2‐yl)‐2,5‐diphenyltetrazolium bromide (MTT; 5 mg/mL; Sigma Aldrich) for 4 h to dissolving blue formazan crystals, the cell viability of three cell lines were measured by spectrophotometry (BioTek) at 563 nm.

### 2.3 Clonogenic assay

The clonogenic assay has described previously.^27^ Briefly, SCC‐9 and HSC‐3 cells were seeded in 6 cm dish in 80–90% confluent, respectively. Various doses of hispolon (25, 50, 75, and 100 μM) or vehicle control were treated after 16 h cells adhesion. After 24 h treatment, 500‐cells of SCC‐9 and HSC‐3 cells were seeded in 6‐well plates for 10 days cell growth, respectively. Fresh medium was replaced every 2–3 days. Finally, colonies were fixed with methanol and stained with 10% Giemsa staining buffer (Sigma Aldrich). Colony numbers of SCC‐9 and HSC‐3 cell lines were captured and counted under a microscope.

### 2.4 Fluorescent nuclear stain assay

SCC‐9 and HSC3‐ cells were seeded and treated different doses (25, 50, 75, and 100 μM) of hispolon or vehicle control for 24 h. Then, the cells were fixed with 4% paraformaldehyde and stained with 50 mg/mL DAPI staining buffer for 5 min, avoiding light. The fluorescence of cells was analysed by Olympus IX73 inverted fluorescence microscope.

### 2.5 Cell apoptosis analysis

The SCC‐9 and HSC‐3 cell lines were seeded in 6‐well plates at 80–90% confluent. Cells were collected after being treated with various doses of hispolon (25, 50, 75, and 100 μM) or vehicle control for 24 h. Each treated group was counted for 1 × 10^5^ cells and incubated with Annexin V and Propidium Iodide (PI) reagents (BD Biosciences) for 20 min avoiding light at room temperature. The stained cells were then measured using BD Accuri C6 Plus Cytometer (BD Biosciences) and analysed with BD CSampler Plus software (version 1.0; BD Biosciences).

### 2.6 Human apoptosis proteome profiler analysis

A membrane‐based antibody Human Apoptosis Array (R&D System) was used to detect 35 apoptosis‐related proteins from SCC‐9 cells after 24 h of hispolon (100 μM) or vehicle treatment. Image‐Pro Plus software has been used to quantify each spot. Spot density is normalized to each reference points, and then normalized to the vehicle control group.

### 2.7 Caspase‐3 detection analysis

For caspase‐3 activated detection, BioTracker™ NucView® 488 Green Caspase‐3 Dye (MilliporeSigma) was used for analysis. After hispolon treatment, SCC‐9 cells were stained with NucView® 488 substrate stock solution (2 μM) for 30 min, avoiding light. Cells can be observed with green fluorescence by Olympus IX73 inverted fluorescence microscope.

### 2.8 Western blot assay

The SCC‐9 and HSC‐3 cell lines were collected after being treated with various doses of hispolon (25, 50, 75, and 100 μM) or vehicle control for 24 h. For Western blot, cells lysates were collected and separated by 10–15% SDS‐polyacrylamide gel electrophoresis (SDS‐PAGE). After electrophoresis, the proteins were subsequently transferred onto PVDF membranes (Millipore) and blocked with 5% skimmed milk for 1 h at room temperature. Indicated primary antibodies, including anti‐PARP, anti‐pro‐caspase‐3, ‐8, 9, anti‐cleaved caspase‐3, ‐8, ‐9, anti‐ERK1/2, anti‐phospho‐ERK1/2, anti‐JNK1/2, anti‐phospho‐JNK1/2, anti‐p38 MAPK, anti‐phospho‐p38 MAPK, anti‐HO‐1, anti‐cIAP1 (Cell Signalling Technology), and anti‐β‐actin (Abcam) were then added to probe the membrane overnight. After washed with Tris‐buffer saline‐0.1% Tween‐20 (TBS‐T) and incubated with secondary antibodies (Seracare; 1:10000 dilution), the signals were detected using Luminescent Image Analyser (LAS 4000 mini, GE Healthcare Bio‐Sciences). The relative photographic density was quantified by Image J.

### 2.9 RNA Interference analysis

Human siRNAs for non‐specific control and HO‐1 were obtained from Ambion Inc. The transfection of siRNA was using Lipofectamine RNAi MAX reagent (Invitrogen) by following a previously described process.^28^, ^29^ At 48 h after transfection, cells were used for HO‐1 detection by Western blotting assay.

### 2.10 Statistical analysis

All results were replicated at least three times and reported as mean ± standard deviation (SD). One‐way analysis of variance with Tukey's honest significant difference was used to examine the variation between control group and treatment groups in all experiments. The present statistical measurements were performed by Sigmaplot v10.0. (Systat Software Inc.). A *p* < 0.05 was regarded as statistically significant.

## 3 RESULTS

### 3.1 Hispolon possess cytotoxicity in OSCC cells

**FIGURE 1:**
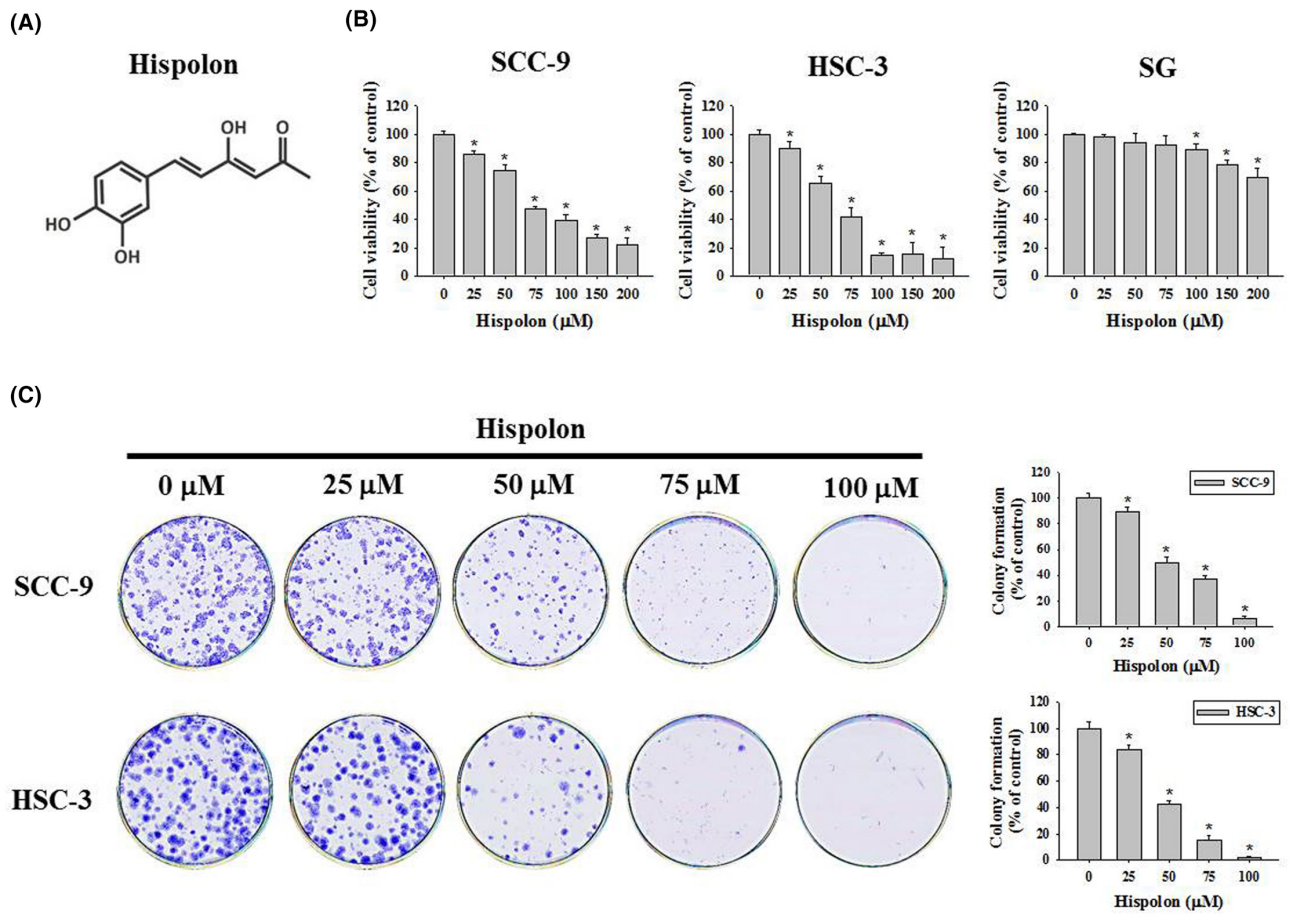
Effect of hispolon on cell viability and colony formation of OSCC cells. (A) The chemical structure of hispolon. (B) OSCC cells, SCC‐9, HSC‐3 or SG cells were treated with hispolon at various doses (25, 50, 75, 100, 150, and 200 μM) or DMSO (vehicle control) for 24 h, and cell survival was determined by the MTT assay. (C) Long‐term cell growth of SCC‐9 and HSC‐3 cells was determined by colony formation after 24 h hispolon (25–100 μM) or vehicle treatment. Data from three independent experiments, each in triplicate, are represented as mean ± SD. *p < 0.05, compared to the vehicle control group.

The chemical structure of hispolon as shown in Figure 1A. To examine the cytotoxicity of hispolon in oral cancer cells, we first compared the cell viability of the hispolon‐treated OSCC cell lines (SCC‐9 and HSC‐3) with the normal gingival epithelial cell line (SG). As shown in Figure 1B, the proliferative rate of OSCC cell lines was significantly reduced after hispolon treatment for 24 h in a dose‐dependent manner (*p* < 0.05). The half maximal inhibitory concentration (IC_50_) of hispolon in OSCC cells was about 55 μM. On the other hand, SG cells have little hispolon‐induced cytotoxicity at a dose of less than 100 μM. The result indicated the specificity of hispolon‐induced cytotoxicity in OSCC cells rather than in normal oral cells. The tumorigenic analysis of the hispolon was then investigated by a colony formation assay. After 10 days treatment, colony formation was visualized by Giemsa staining, which showed suppressing in hispolon treatment groups (25, 50, 75, and 100 μM) in both SCC‐9 and HSC‐3 cells (*p* < 0.05) (Figure 1C). The IC50 values of SCC‐9 and HSC‐3 were less than 50 μM in clonogenic assay. The results indicated that hispolon not only has short term cytotoxicity in OSCC cells but also showed anti‐tumorigenic effect on long term experiment.

### 3.2 Hispolon trigger programmed cell death of OSCC cells

**FIGURE 2:**
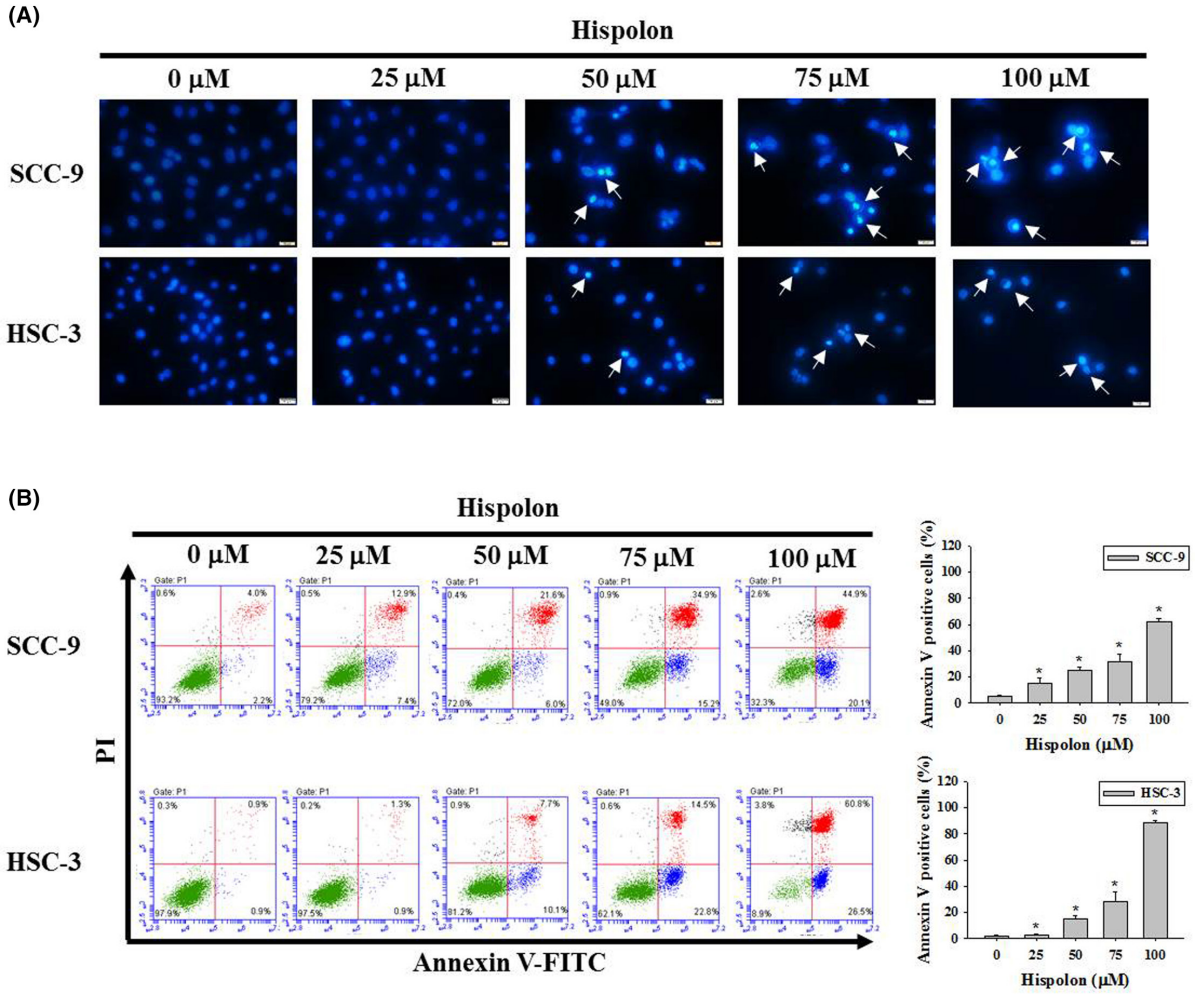
Effect of hispolon on cell apoptosis observed by DAPI and annexin V–FITC/PI staining. (A) SCC‐9 and HSC‐3 cells were treated with hispolon at various doses (25–100 μM) for 24 h. The chromatin condensation was observed by fluorescence microscopy after DAPI staining. The arrow represents fragmented and condensed nuclei as indicated apoptotic cells. (B) After 24 h of hispolon treatment (25–100 μM) or vehicle treatment of SCC‐9 and HSC‐3 cells, early and late apoptotic cells were determined by flow cytometric analysis and quantified. Data from three independent experiments, each in triplicate, are represented as mean ± SD. *p < 0.05, compared to the vehicle control group.

The morphological markers of apoptosis, including chromatin condensation and phosphatidylserine (PS) exposure, were then examined using DAPI and annexin V/PI stained, respectively. The condensed of DNA was found to increase after treatment with hispolon in both SCC‐9 and HSC‐3 cell lines (Figure 2A, arrows). With the combination of PS and annexin V‐FITC pointed out early apoptosis and late apoptosis cells that were induced by hispolon treatment for 24 h in OSCC cells (Figure 2B). These characteristics of apoptosis indicate that hispolon have the ability to induce apoptosis of OSCC cells.

### 3.3 Hispolon increases HO‐1 expression and induces caspase‐mediated apoptotic cell death of OSCC cells

**FIGURE 3:**
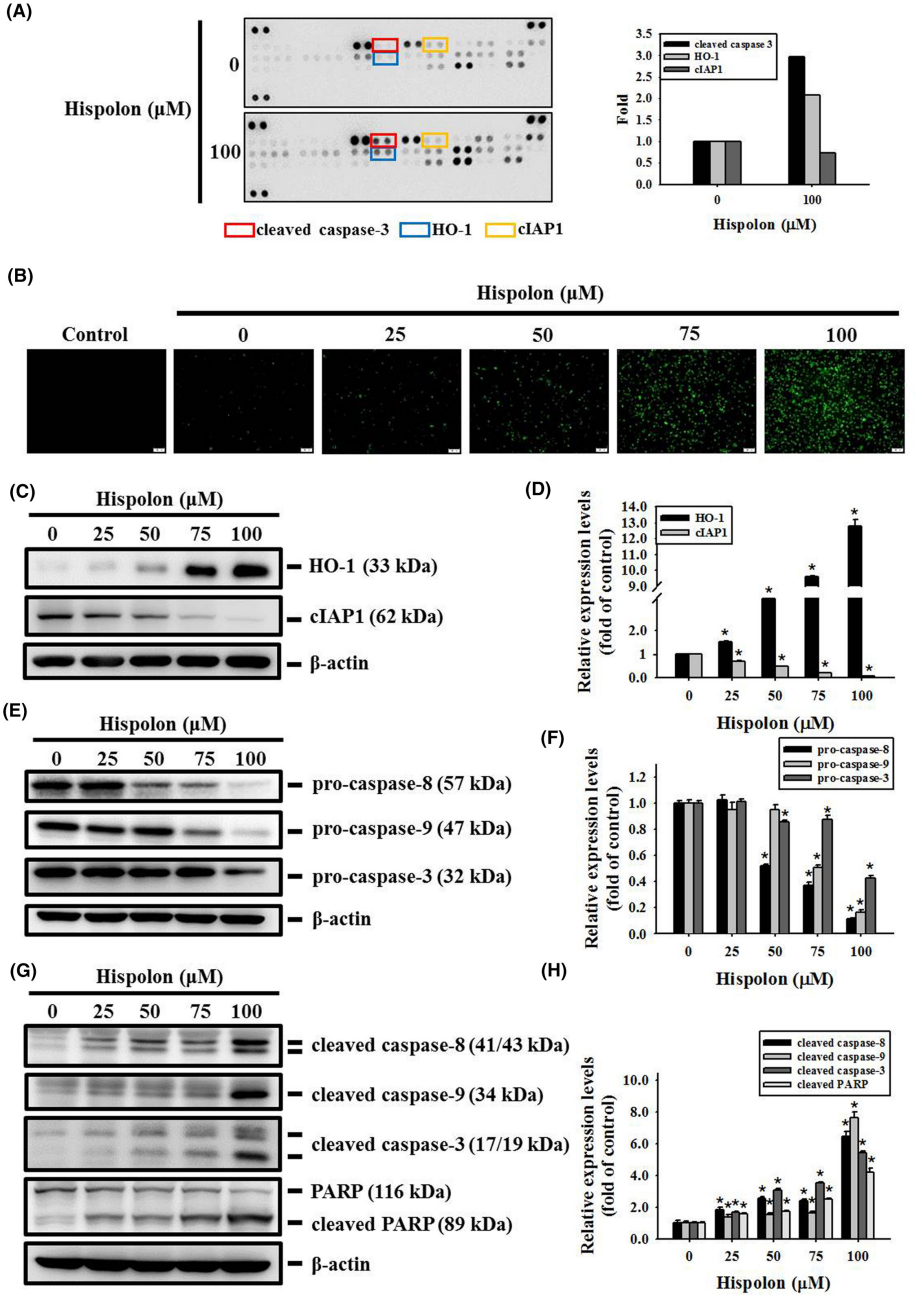
Effect of hispolon on apoptosis‐related proteins of OSCC cells. (A) A membrane‐based antibody Human Apoptosis Array (R&D System) was used to detect 35 apoptosis‐related proteins from SCC‐9 cells after 24 h of hispolon (100 μM) or vehicle treatment. Scanned images of Human Apoptosis Array were quantified with a densitometer and expressions relative to the control group. (B) Representative images and analysis of Nucview 488 Green Caspase‐3 Dye staining of SCC‐9 cells after 24 h of hispolon (25–100 μM) or vehicle treatment were analysed by fluorescence microscopy. (scale bar: 100 μm) (C–H) Protein expression level of related regulators (HO‐1, cIAP1, pro‐ and cleaved caspases‐3, ‐8 and ‐9, and PARP) in the apoptosis pathways after 24 h hispolon treatment (25–100 μM) or vehicle treatment were confirmed by Western blots and quantified by Image J. Normalisation of protein levels is performed using β‐actin to accurately quantify Western blots. Data from three independent experiments, each in triplicate, are represented as mean ± SD. *p < 0.05, compared to the vehicle control group.

To clarify the apoptosis‐related proteins that are regulated by hispolon in OSCC cells, we execute a proteome profiler human apoptosis array with the SCC‐9 cell line. Compared to vehicle and hispolon at a dose of 100 μM, the protein level of cleaved caspase‐3 and heme oxygenase‐1 (HO‐1) were increased while the cellular inhibitor of apoptosis protein‐1 (cIAP‐1) was decreased after hispolon treatment (Figure 3A). Using caspase‐3 detection analysis, activated caspase‐3 was found to increase with green fluorescence after treatment with different doses of hispolon (25, 50, 75 and 100 μM) (Figure 3B). Similar to Figure 3A, the overexpression of HO‐1 and suppression of cIAP1 were found in a dose‐dependent manner of SCC‐9 cells after hispolon treatment. (Figure 3C,D). Regarding the key initiator of the extrinsic and intrinsic pathway of apoptosis, hispolon treatment reduced pro‐casapse‐3, ‐8 and ‐9 in the SCC‐9 cell line (Figure 3E,F). Meanwhile, activated caspases such as cleaved caspase‐3, ‐8 and ‐9 were found elevated, as well as cleaved poly ADP‐ribose polymerase (PARP) (Figure 3G,H). These results indicate that hispolon can stimulate OSCC cell apoptosis possibly through regulation of HO‐1, cIAP1, caspases and PARP.

### 3.4 HO‐1 involves in hispolon‐regulated apoptosis as an upstream regulator of OSCC cells

**FIGURE 4:**
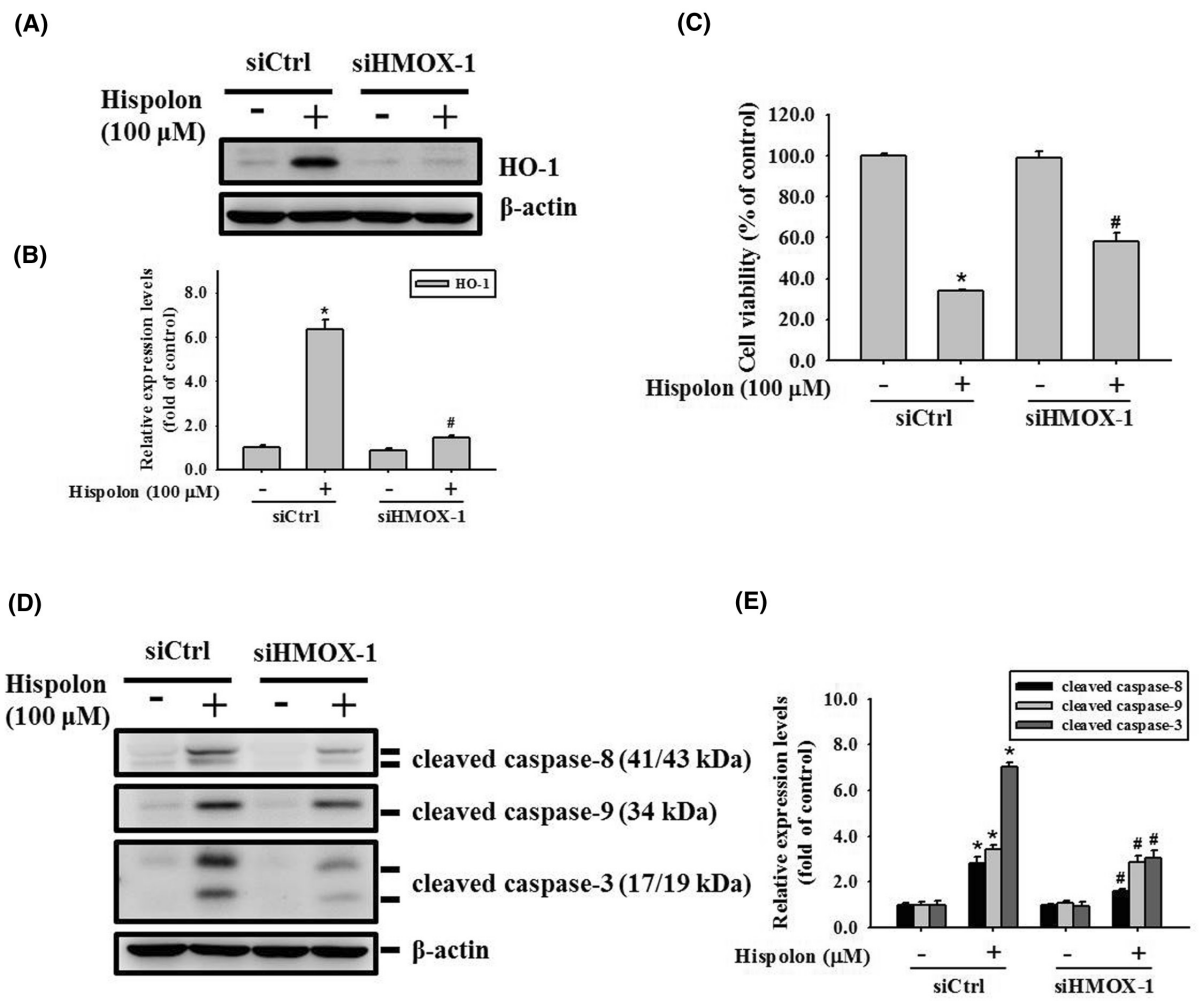
Involvement of HO‐1 in hispolon‐induced apoptosis in OSCC cells. (A) HO‐1 was knocked down by transient transfection of *HOMX1* siRNA (si*HOMX1*) or control siRNA (siCtrl) in SCC‐9 cells with or without Hispolon (100 μM) treatment. The Western blot analysis showed the knockdown efficiency of *HOMX1* siRNA. Quantitative results of HO‐1 protein levels are shown in (B). (C) The MTT assay was used to evaluate the combination effect of *HOMX1* siRNA and hispolon (100 μM) treatment on cell viability. (D) The protein expression level of apoptosis‐related proteins (cleaved caspases‐3, ‐8, and ‐9) after the combination of *HOMX1* siRNA and hispolon (100 μM) treatment were confirmed by Western blots. Quantitative results of protein levels are shown in (E). Normalisation of protein levels is performed using β‐Actin to accurately quantify Western blots. Data from three independent experiments, each in triplicate, are represented as mean ± SD. *p < 0.05, compared to the vehicle control group; #*p* < 0.05, compared to the siCtrl‐transfected group.

Elevated HO‐1 has been reported to be involved in the apoptosis process in OSCC cells.^30^, ^31^ To understand the role of HO‐1 in hispolon‐induced apoptosis, the HO‐1 RNA interference system is applied in subsequent experiments. First, we detected the HO‐1 RNA interference system and confirmed that the hispolon‐induced HO‐1 protein level is down‐regulated by treating with HO‐1 siRNA (Figure 4A,B). Subsequently, as shown in Figure 4C, hispolon‐induced cytotoxicity has been rescued in the HO‐1 siRNA treatment group compared to the control siRNA group. Meanwhile, Western blot analysis illustrated that the protein level of cleaved caspase‐3, ‐8 and ‐9 which activated by hispolon was suppressed after HO‐1 siRNA treatment (Figure 4D,E). The above data reveal that HO‐1 is involved in hispolon‐regulated apoptosis in SCC‐9 cells.

### 3.5 Hispolon activates JNK/HO‐1 signalling cascade in caspase‐mediated apoptotic cell death of OSCC cells

**FIGURE 5:**
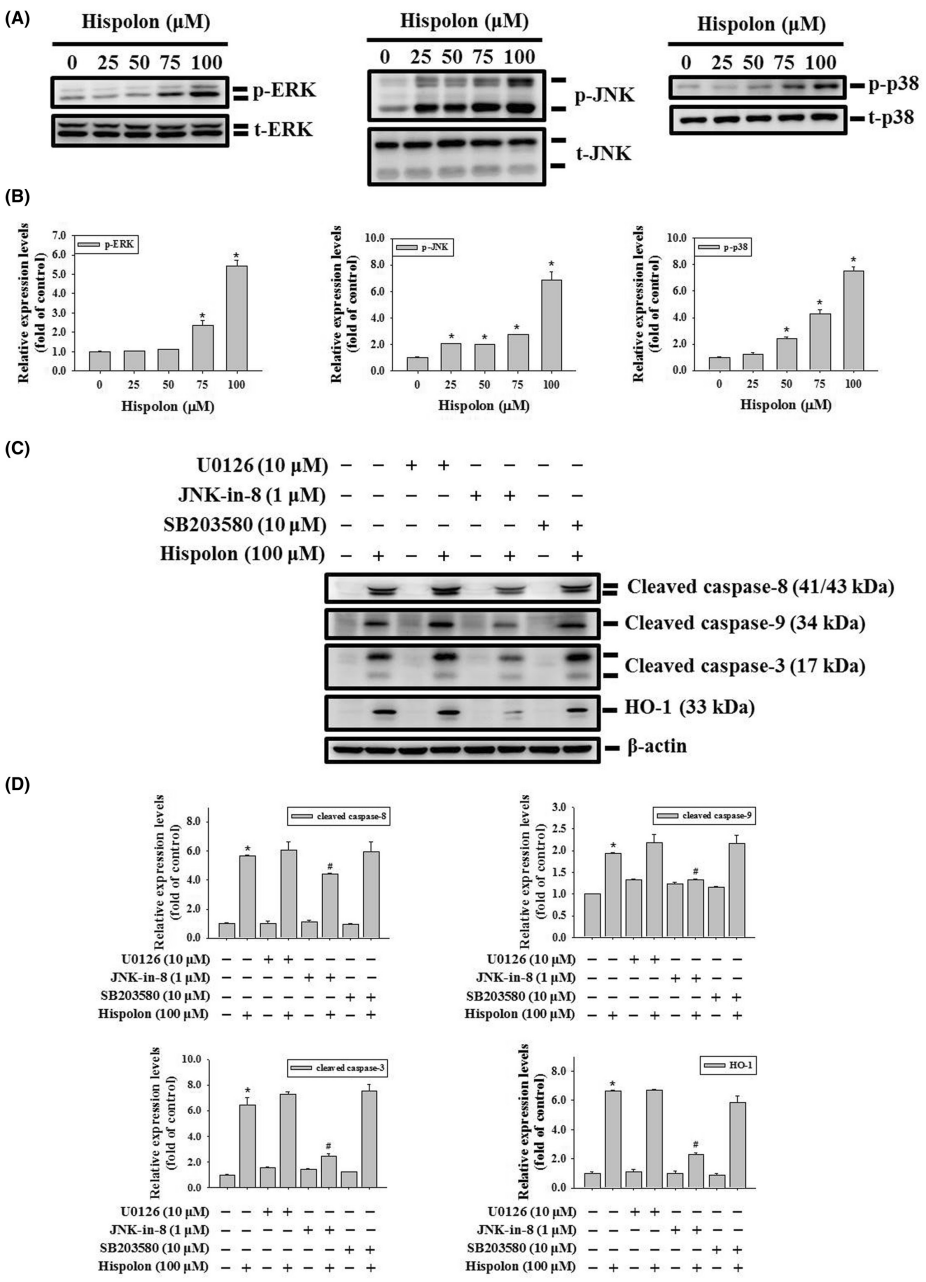
The JNK1/2 signalling pathway is involved in hispolon‐mediated HO‐1 overexpression and cell apoptosis. (A) The protein expression levels of ERK1/2, p38, and JNK1/2 were detected by Western blot analysis with hispolon treatment (25–100 μM) for 6 h in SCC‐9 cells. (B) Normalisation of phospho‐MAPKs levels is performed using total MAPKs level to accurately quantify western blots. (C) SCC‐9 cells were pretreated with U0126, JNK‐in‐8 or SB203580 for 1 h followed by vehicle or hispolon treatment (100 μM) treatment for an additional 24 h. The expression levels of cleaved caspase‐3, ‐8 and ‐9 protein and HO‐1 were detected by a Western blot analysis. Quantitative results of protein levels are shown in (D). Normalisation of protein levels is performed using β‐actin to accurately quantify Western blots. Data from three independent experiments, each in triplicate, are represented as mean ± SD. *p < 0.05, compared to the vehicle control group; # *p* < 0.05, compared to the hispolon‐treated group.

**FIGURE 6:**
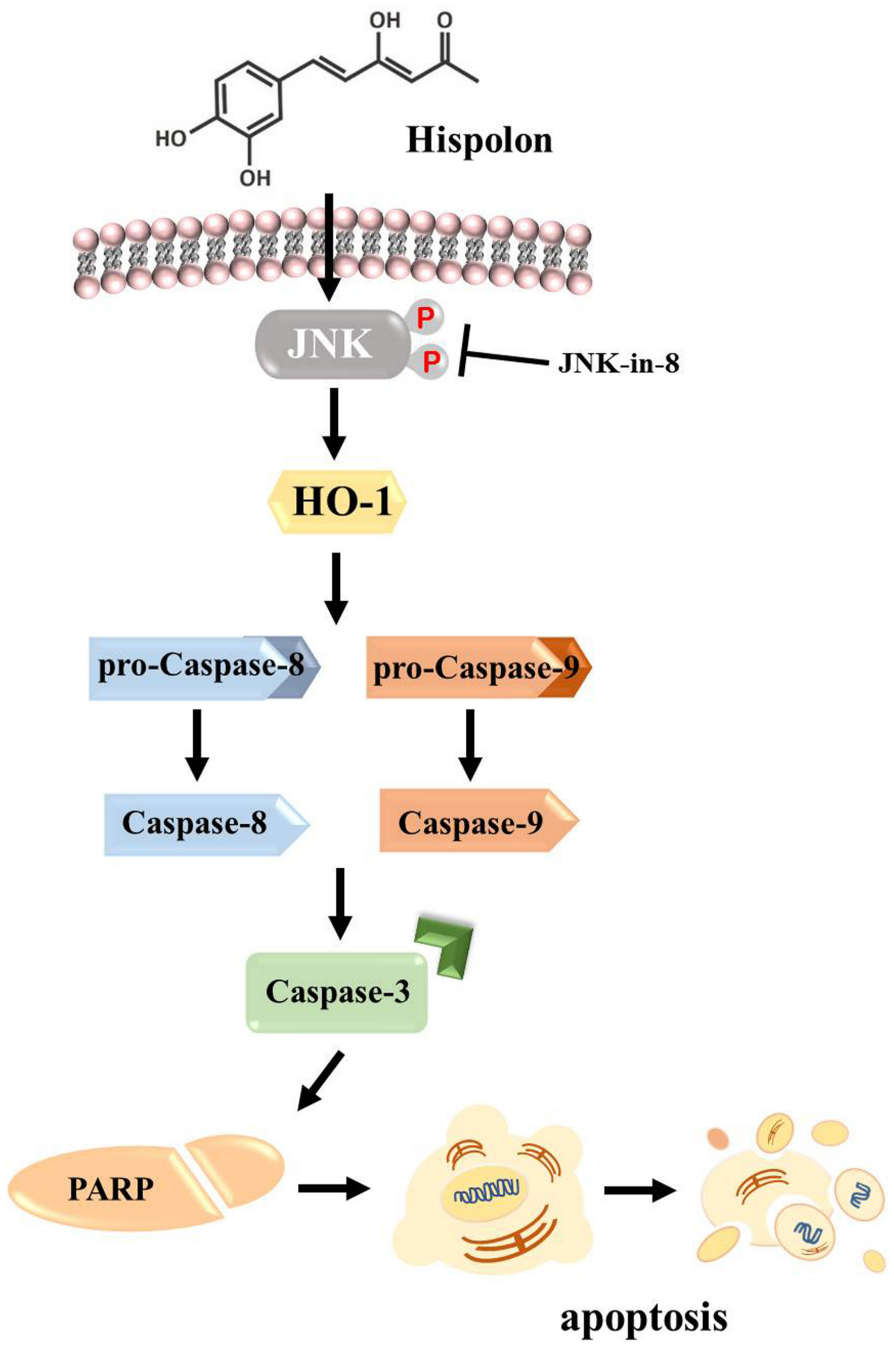
Schematic diagram model of the molecular mechanism on anticancer effects of hispolon of OSCC cells. Hispolon can induce caspase‐mediated apoptotic cell death through the JNK/HO‐1 pathway in OSCC cells.

Mitogen‐activated protein kinase (MAPK) pathways are signalling cascades that connect the signal from the extracellular to the nucleus and regulate biological processes such as cell proliferation, cell differentiation and cell death.^32^ To investigate the role of the MAPK pathway in hispolon‐regulated apoptosis, we examined the activated form of MAPK proteins, phosphor‐extracellular signal‐regulated kinase (p‐ERK)1/2, p‐Jun kinase (JNK)1/2, and p‐p38 using Western blot analysis. As shown in Figure 5A,B, p‐ERK1/2, p‐JNK1/2 and, p‐p38 protein expression was significantly up‐regulated after hispolon treatment in a dose‐dependent manner. Subsequently, we added MAPK inhibitors, U0126 (ERK inhibitor), JNK‐in‐8 (JNK inhibitor) and SB203580 (p38 inhibitor) in the experiment to further elucidate the pathway that directly involved in hispolon regulation. Compared with hispolon alone, cleaved caspase‐3, −8, −9 and HO‐1 were suppressed in a group that cotreated with hispolon and JNK in 8 (Figure 5C,D). However, the cleaved caspase‐3, ‐8, ‐9 and HO‐1 remained unchanged in hispolon combined with U0126 or SB203580. These results indicate that hispolon‐regulated apoptosis via the JNK1/2 pathway in SCC‐9 cells. Taken together, our results confirmed the underlying mechanism of hispolon in OSCC cells that hispolon‐induced apoptotic cell death is regulated by cleaved caspase‐3, ‐8 and ‐9, and HO‐1 activation through the JNK signalling pathway (Figure 6).

## 4 DISCUSSION

Hispolon, a bioactive constituent of traditionally used medicinal mushrooms, has been reported against 13 different types of cancer to date.^22^ Due to the broad range of medicinal properties, we aimed to investigate the chemotherapeutic effect of hispolon on OSCC cells. In present the study, we demonstrated that hispolon lead to high cytotoxicity of the OCSS cell lines SCC‐9 and HSC‐3, but not the human gingival epithelial cell line SG. The mechanism of hispolon‐mediated apoptosis is associated with the activation of cleaved caspase‐3, ‐8 ‐9, and PARP. Moreover, our results showed that hispolon induces OSCC cell apoptosis through the elevation of HO‐1 mediated by the JNK pathway. These data supported that the hispolon is a potential agent for OSCC treatment.

Apoptosis is beneficial to cancer therapy, which can be recognized with its characteristic morphological changes, including chromatin condensation, nuclear fragmentation, membrane blebbing and cell shrinkage.^33^ Previous studies had reported that chromatin condensation and annexin V positive cells were increased after hispolon treatment in nasopharyngeal carcinoma cells^34^ and hepatocellular carcinoma cells,^23^ which were consistent with our results. As a crucial role that participated in the initiation of apoptosis, caspases can be active by the death receptor pathway and the mitochondrial pathway (also known as extrinsic pathway and intrinsic pathway, respectively).^35^ The combination of Fas or tumour necrosis factor (TNF) —related apoptosis‐inducing ligand (TRAIL) receptors with Fas ligand or TRAIL caused Fas‐associated death domain (FADD) and caspase‐8 recruitment and triggers caspase‐3 activation.^36^ On the other hand, the release of cytochrome c from mitochondria initiated apoptosome complex formation (including cytochrome c, Apaf‐1 and caspase‐9), which also activated caspase‐3 and caused downstream processes of apoptosis.^37^ In the present study, caspase‐9, ‐8 and ‐3 were activated after hispolon treatment in both SCC‐9 and HSC‐3 cells. These findings confirm those of earlier studies, such as hispolon caused cleaved caspase‐3, ‐8 and ‐9 increasing in acute myeloid leukaemia^38^ and gastric cancer cells.^39^

The inhibitor of apoptosis proteins (IAPs) family members are represented a group of endogenic negative modulator of caspases, which are frequently overexpressed in tumour cells and promoted chemo‐resistance and tumour survival.^40^ Among the IAPs family, cIAP1 has been shown to suppress the pro‐caspase‐3 activation by interacting with apoptosome complex.^41^ Furthermore, Zhang's study indicated that cIAP1/2 functionally suppressed caspase‐8‐dependent cell death in vivo.^42^ Similar to the above findings, our present data demonstrated that the down‐regulation of cIAP1 and up‐regulation of caspase‐3, ‐8 and ‐9 were found in hispolon‐induced cell apoptosis.

Heme oxygenase (HO) is a metabolic enzyme that generates carbon monoxide (CO), biliverdin, and iron in the rate limitation step of heme degradation.^43^ Two main HO isoforms, HO‐1 (inducible isozyme) and HO‐2 (constitutively express isozyme), exert cellular HO activity with different biochemical properties.^44^, ^45^ A hypothesis has mentioned the noncanonical role of HO‐1, which may transcend the catalytic of heme, and participating cell death regulation, such as modulating cellular autophagy, apoptosis, ferroptosis or pyroptosis programs.^46^ Interestingly, previous reports showed that HO‐1 may play a dual role in apoptosis among different types of cancer. The elevation of HO‐1 is supposed to promote tumour cell proliferation and survival, such as in gastrointestinal tumours,^47^ non‐small cell lung cancer,^48^ and prostate cancer.^49^ Nevertheless, up‐regulation of HO‐1 is also found in drug‐mediated tumour apoptosis, especially in head and neck squamous cell carcinoma. Our previous studies have indicated that curcumin analogues increased HO‐1 expression in OSCC apoptosis through MAPK pathways,^28^, ^29^ which agrees with our present study that HO‐1 is overexpressed in hispolon‐induced apoptosis.

Among apoptosis, the MAPKs signalling pathway can be an activator or inhibitor, depending on different stimulus and cell types. Huang et al. have reported that hispolon‐induced caspase‐mediated apoptosis through inhibition of ERK in hepatocellular carcinoma.^23^ Additionally, hispolon has been found to promote the activation of ERK1/2, JNK1/2, and p38 in cells induced by apoptosis of nasopharyngeal carcinoma.^34^ Similar results were obtained in the present study that p‐ERK1/2, p‐JNK1/2, and p‐p38 increased after hispolon treatment. However, the combination of hispolon and MAPK inhibitor confirmed that the JNK pathway is the main pathway which involved in hispolon‐regulated apoptosis in OSCC cells.

It is worth noting that the apoptotic effect of hispolon has been recorded to result in loss of mitochondrial membrane potential and caspase‐3 activation in the KB cell line.^50^ Although the KB cell line was established from squamous cell carcinoma of the larynx, the report had demonstrated that KB cells were contaminated by HeLa cells.^51^ Based on the literature, the present results become reliable evidence that demonstrated the underlying mechanism of hispolon in OSCC cells.

Furthermore, the IC_50_ of hispolon has been reported at a concentration below 100 μM in various types of cancer, such as lung cancer (A549 cell line IC_50_ = 35.9 ± 6.9 μM),^52^ prostate cancer (DU145 cell line IC_50_ = 31 μM),^53^ and acute myeloid leukaemia (IC_50_ = 6.25–25 μM).^38^ The present result also showed the hispolon sensitivity of the OSCC cell lines SCC‐9 (IC_50_ = 58.24 μM) and HSC‐3 (IC_50_ = 55.81 μM). Interestingly, the MTT assay demonstrated that hispolon was less sensitive to normal gingival epithelial cells than OSCC cells. On the basis of our finding, it might consider hispolon for the pharmacological application in OSCC patients. Considering the limited of not using animal model in present study, further research is needed to verify the ability of hispolon on anti‐tumour growth in vivo. Moreover, the anticancer activity of hispolon with 3D cell culture model also needs to be further investigated in the future.

Taken together, our study has provided mechanistic understanding of the hispolon apoptotic machinery on the regulation of JNK/HO‐1/caspase in OSCC cells. We merit that our results can provide evidence on the potential of hispolon as specific chemotherapeutic agent for OSCC.

## AUTHOR CONTRIBUTIONS

**Wei‐En Yang:** Conceptualization (equal); writing – original draft (equal); writing – review and editing (equal). **Yi‐Tzu Chen:** Writing – original draft (equal). **Chun‐Wen Su:** Methodology (equal). **Mu‐Kuan Chen:** Conceptualization (equal). **Chia‐Ming Yeh:** Methodology (equal). **Yen‐Lin Chen:** Methodology (equal). **Meng‐Ying Tsai:** Methodology (equal). **Shun‐Fa Yang:** Conceptualization (equal); writing – original draft (equal); writing – review and editing (equal). **Chiao‐Wen Lin:** Conceptualization (equal); writing – original draft (equal); writing – review and editing (equal).

## FUNDING INFORMATION

This study was supported by Chung Shan Medical University Hospital, Taiwan (CSH‐2022‐D‐003).

## CONFLICT OF INTEREST STATEMENT

The authors declare that there is no conflict of interest.

## Data Availability

The data used to support the findings of the present study are available from the corresponding author upon request.
